# Global Health diplomacy for noncommunicable diseases prevention and control: a systematic review

**DOI:** 10.1186/s12992-020-00572-5

**Published:** 2020-05-06

**Authors:** Mahnaz Afshari, Ahmad Ahmadi Teymourlouy, Mohsen Asadi-Lari, Mohammadreza Maleki

**Affiliations:** 1 Department of Health Service Management, School of Health Management and Information Sciences, Iran University of Medical Sciences, Tehran, Iran; 2 Department of Epidemiology, School of Public Health, Iran University of Medical Sciences, Tehran, Iran

**Keywords:** Review [publication type], Diplomacy, Global Health, International cooperation, Noncommunicable diseases, Policymaking

## Abstract

**Introduction:**

The prevention and control of noncommunicable diseases (NCDs) are one of the main challenges of healthcare systems around the world. In addition to the technical level, it requires political negotiations and solutions, such as global health diplomacy (GHD), which involves the participation of a wide range of actors and stakeholders and innovative international health partnerships. This review aimed to draw lessons for strengthening linkages with a wide range of actors and stakeholders from the GHD literature for NCDs, and how policymakers and political leaders can effectively use international health partnerships to beat NCDs.

**Methods:**

This research was a systematic review of the literature on GHD for NCDs. All relevant articles published in English were identified by searching PubMed, Web of Science, Scopus, and Embase databases, Google and Google Scholar search engines, and the reference lists of identified articles as well as a number of special journals. 30 articles that met the inclusion criteria were analyzed using content analysis in MAXQDA 10. The Global Health Diplomacy Pyramid and Blouin and Dubé’s (2010) analytical framework for examining negotiations were used to classify the data.

**Findings:**

30 articles have been published on GHD for NCDs. Five key themes, i.e. the specific problem requiring global collective action, key actors, their interests in the problem, potential negotiation process, and potential scenarios for collective action and 46 sub-themes were identified. Moreover, given the importance of collaboration on NCDs in the international arena, actors were categorized into three groups based on the GHD Pyramid: (1) core diplomacy, (2) multi-stakeholder diplomacy, and (3) informal diplomacy.

**Conclusion:**

Development and adoption of a global policy to tackle the rise in NCDs in developed and developing countries require policymakers and political leaders that participate in GHD. Successful developments in global health policy depend on the performance of and respectful relationships among the stakeholders, and global health diplomats need to understand the complexities of the institutional structures and functional relationships of the international institutions involved in health.

## Introduction

Over the last two decades, global health issues have become more prominent in foreign policy. Events such as the HIV/AIDS pandemic, infectious diseases, the threat of bioterrorism, and issues related to trade and health have prompted policymakers to focus more on health issues [1–3]. As nations become more and more interconnected and as health-related issues increasingly become global issues, state actors in the domain of health are forced to seek cross-border collective action and collaboration with nonstate actors [2, 4]. United Nations (UN) General Assembly also emphasizes that governments must pay more attention to global health in their foreign policies and increase their negotiations and political interactions in this field [5–7].

Global health diplomacy (GHD) is the process of negotiated collective action for global health that can eventually lead to new forms of global health policy and governance [8]. This concept has received significant attention from key global health actors, including the World Health Organization (WHO), health and foreign affairs ministries of countries, and academia [9–13].

Although GHD has entered the mainstream of countries’ foreign policy, it has different meanings, which could generally be divided into three categories based on the interaction of actors around global public health issues: (1) core diplomacy, i.e. formal negotiations among nations; (2) multi-stakeholder diplomacy, i.e. negotiations among nations and other actors that do not necessarily lead to agreements; and (3) informal diplomacy, i.e. interactions between international public health actors and their counterparts, including host country officials, nongovernmental organizations, private companies, and the public. Core and multi-stakeholder diplomacy in global health require the effective use of a delicate mix of technical expertise, legal knowledge, and diplomatic skills [14]. A successful global health strategy for addressing public health and foreign policy goals requires effective action at every level of the GHD pyramid, and GHD, as practiced by health attachés, entails the identification and engagement of key tools and actors as well as coordinated action by various counterparts and stakeholders [15].

Health diplomacy provides a political framework that aims to improve health in target populations and enhance governmental relations between collaborating countries. Governments offering health-related aid to a nation with which they wish to develop stronger diplomatic links have the advantage of developing a deeper relationship with its citizens [16]. This can be accomplished through different mechanisms such as providing general funding, ensuring the supply of essential drugs, investing in hospitals or equipment, and training health professionals. Significant investments have been made in Sub-Saharan Africa, South America, and South-East Asia to eradicate specific diseases such as HIV/AIDS, malaria, and tuberculosis. However, there has been relatively little support for general health infrastructure, training, and education, or the burden of chronic diseases in developing countries [17].

Each year, 41 million people die from noncommunicable diseases (NCDs), which is equivalent to 71% of all deaths globally [18]. Evidence suggests the increasing burden of NCDs in low and middle-income countries (LMIC) [19, 20]. NCDs in LMIC account for 80% of deaths and two-thirds of disabilities from NCDs worldwide. More specifically, NCDs in LMIC mostly affects people in their thirties, which is their most productive working years and this is serious threat to health and economic growth [21].

There is widespread international support for the fight against NCDs. The UN High-Level Meeting on Prevention and Control of NCDs [22], the WHO Global Action Plan for the Prevention and Control of NCDs 2013–2020 [23, 24], the WHO NCD Global Monitoring Framework [25], and recognition of NCDs as a major challenge to sustainable development [26] highlight this issue. Although such support for prevention and control of NCDs is encouraging, lack of international financing for these efforts necessitates the development of multi-stakeholder models to address the global burden of NCDs.

Prevention and control of NCDs is one of the key challenges of health systems that, in addition to a technical level, requires negotiations and political solutions such as global health diplomacy (GHD), which involves the participation of a wide range of actors and stakeholders [27]. Policy interventions for health, prevention, and control of NCDs must begin with diplomatic negotiations between state officials [28]. Health threats such as challenges to the safety of the global drug supply and the spread of chronic and NCDsthat impact national security increasingly highlight the need for diplomats who understand health issues and can negotiate effectively in the multinational foreign policy environment [27–29]. Insufficient focus on NCDs by different sectors at the national and international levels, including the lack of funding for NCD research, prevention, and control by governments and non-governmental organizations (NGOs) necessitate the development of coordinated strategies and diplomatic initiatives to address the multinational nature of this issue [30]. GHD for NCDs is defined as the process of negotiations by which state and nonstate actors attempt to develop and implement collective actions to solve global health challenges [1–4]. This paper argues that global health diplomacy and political interactions and partnerships between policy actors could contribute to a more effective global response and action to NCDs and leading to global health improvements. The purpose of this review is to draw lessons from the GHD literature for NCDs and how it can be effectively used by policymakers and political leaders.

## Methods

This research ia a systematic review of the existing evidence on GHD for NCDs, including cardiovascular diseases, cancers, chronic respiratory diseases, and diabetes as well as their risk factors, i.e. tobacco use, excess salt and fat intake, alcohol abuse, and physical inactivity, in the period 2007–2019. The second stage involved content analysis of the studies with a qualitative approach.

To find relevant articles for this review, PubMed, Web of Science, Scopus, and Embase databases and Google and Google Scholar search engines were used. Keywords included MeSH and common terms related to the topic: “Diplomacy” OR “Internationality” OR “foreign policy” OR “foreign affairs” OR “international relations” OR “international politics” OR “statesmanship” OR “statecraft” OR “Health Diplomacy” OR “Medical Diplomacy” OR “Negotiations” OR “Multilateral Engagement” OR “Bilateral Agreements” AND “Drinking Behavior” “Alcoholic Beverages” OR “Smoking” OR “Smokers” OR “Feeding Behavior” OR “Diet” [MeSH] OR “Obesity” OR “Food” OR “Fast Foods” OR “Sugars” OR “Sodium, Dietary” OR “Exercise” [MeSH] OR “LifeStyle” OR “Healthy Lifestyle” OR “Sedentary Behavior” OR “Alcohol Drinking” [MeSH] OR “Tobacco Use” OR “Tobacco Products” OR “Tobacco” OR “Noncommunicable Diseases”. Moreover, the reference lists of identified articles were manually searched to find more relevant studies. The studies included in the qualitative synthesis include empirical studies, commentaries and review articles. Inclusion criteria for this study included literatures published in English language and the year of publication included studies published from 2007 to 2019 because initial searches of the literature showed that most relevant studies were conducted after 2007. Articles published in any language other than English, articles that were not unavailable in full text, dissertations, and redundant studies were excluded.

**Fig. 1:**
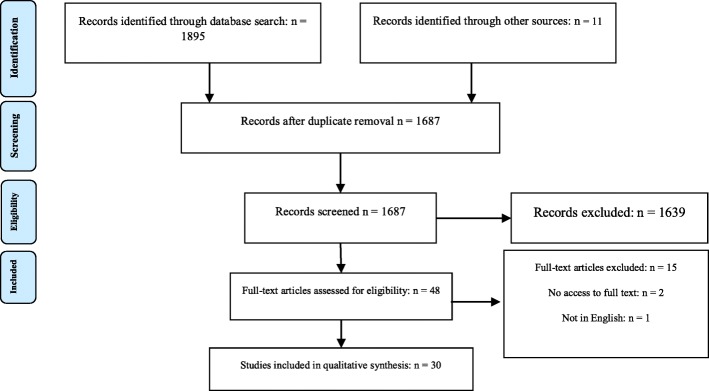
PRISMA Flow Diagram: Database search and article selection process

Overall, 1895 articles were extracted. First, the title and abstract of the articles were screened based on the inclusion criteria and Studies which did not address the research question and the duplicates of the same records were then excluded in this stage and leaving 48 articles for full-text review. 18 articles were excluded due to unavailability in full-text form or redundancy, and 30 articles were selected for the final review. The screening process and search results are provided in the PRISMA flow diagram [31] (Fig. 1). Data were collected using a data extraction form that was designed based on the objectives of the study. This form included entries on the author, publication year, country, method of research, key actors, content, context, Tools and main finding.

The remaining articles were entered into the quality assessment stage. Quality was assessed independently by two studies using the 15-point instrument of Mitton et al. [32]. Each item is given a score of 0 (not present or reported), 1 (present but low quality), 2 (present and midrange quality), or 3 (present and high quality). Criteria for quality assessment included: literature review and identification of research gaps; research questions, hypotheses, and design; population and sampling; data collection process and instruments; and analysis and reporting of results. Disagreements were resolved by discussion and, when essential, by consulting a third review author. Given that the review was qualitative, articles were not removed at this stage, but more weight was given to articles with a quality rating of 10 or above in the data analysis and interpretation of results.

Then, a content analysis of the articles was performed. The data were coded and managed using MAXQDA 10 for Windows (VERBI GmbH, Berlin, Germany), and related themes and sub-themes for each article were extracted to identify the relationships among these themes and identify patterns and meanings. The GHD Pyramid [14, 15] and the Negotiations Review Analytical Framework [4] were used to classify the data.

## Results

**Fig. 2:**
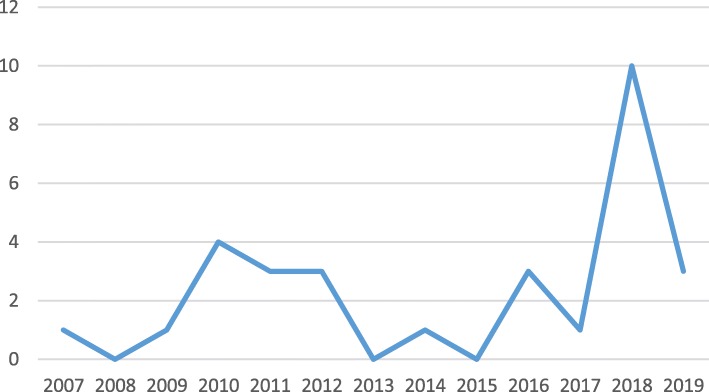
Frequency distribution of studies on GHD for NCDs

Between 2007 and 2019, 30 articles have been published on GHD for NCDs (Table 1). About 57% of the articles have been published since 2016, with the majority of studies (10 articles) published in 2018 (Fig. 2). Trends suggest that the number of articles has increased around 2011 and 2018 in which the UN General Assembly held high-level meetings on the prevention and control of NCDs.

**Table 1:** Characteristics of studies on GHD for NCDs

Author	Country/Region	Year	Journal	Level of Diplomacy	Domain	article type	Method	key findings
Hospedales et al. [33]	CARICOM*	2011	Pan American Journal of Public Health	Regional	Advocacy, Political Factors	Commentary and perspectives	Qualitative- opinion piece on the process of and lessons learned in achieving the political commitment of heads of government manifested by a one-day summit on the prevention and control of NCDs*	Implementation of the NCD Summit Declaration mandates was most effective in larger countries with greater capacity, but countries of all sizes performed well, when they had regional or global support. Progress was limited in regional approaches to food security, labeling, and elimination of trans fats. Inadequate funding stymied several resource-dependent interventions. Monitoring mechanisms were established, but more concrete goals are needed, especially for actions of non-health government agencies*.*
Lencucha et al. [34]	Canada	2010	Health Policy and Planning	Global	NGOs, GHD, FCTC, International Negotiations, Global Health Governance	Original study	Grounded theory—qualitative data from public documents and in-depth interviews with participants from the government and NGOs*	Five key activities or roles of the Canadian NGOs during the negotiation of the FCTC*: monitoring, lobbying, brokering knowledge, offering technical expertise and fostering inclusion.
Blouin [30]	–	2012	Administrative Sciences	Global	Global Health, Diplomacy, NCDs, Chronic Diseases, Policy, Global Collective Action	Review	Review—social sciences literature on policymaking at the domestic and international level	Adopting a global strategy with partners to increase media coverage. key role of civil society organizations in a strong collective response
Lee [35]	Brazil	2010	PLoS Medicine	National/ Global	NGOs, GHD*, FCTC, International Negotiations	Original study	key informant interviews with Brazilian policymakers, diplomats, and public health advocates on the country’s role in FCTC negotiations, literature review of priary and secondary sources	providing leadership throughout the negotiation process
Blouin & Dubé [36]	–	2010	Journal of Public Health Policy	Global	Obesity Prevention, NGOs, GHD, FCTC, International Negotiations	Review	Review of documents and studies— a simple analytical framework is used: (1) the specific problem requiring global collective action, (2) key actors, (3) their interests in the problem, (4) potential negotiation process, and (5) potential scenarios for collective action	require a much stronger engagement with developing countries
Mamudu & Glantz [37]	–	2009	Global Public Health	Global	Civil Society, GHD, FCTC, International Negotiations	Original study	Interview and triangulation of archival documents and	Using proper strategies including publication of a newsletter, shaming, symbolism and media advocacy by the FCA to influence policy positions of countries during the FCTC negotiation.
Teixeira et al. [38]	Brazil	2017	Cadernos de Saúde Pública	National/ Global	Civil Society, GHD, FCTC, International Negotiations	Original study	Kingdon’s perspective	The link between tobacco-related healthcare measures by technically skilled officials, the involvement of the high echelon of the Ministry of Foreign Affairs (policy flow), the initiative for the establishment of the WHO*-FCTC (problem flow), and the existence of a favorable environment in executive and legislative (political flow), opened a opportunity window for WHO-FCTC approval and its inclusion in the government decision agenda.
Sener [39]	–	2014	American Journal of Surgery	Global	Medical Diplomacy, Tobacco Control, Breast Health	Commentary and perspectives	Qualitative	Unprecedented presence and participation of NGOs in the drafting stages was helpful for starting negotiations for the treaty
Smith & Irwin [40]	India	2016	Globalization and Health	National/ Global	Food and Non-alcoholic Beverages to Children, GHD, International Negotiations	Commentary and perspectives	Ethnographic study, in-depth interviews	a successful GHD in international level is part of a process, not the end, and an important part of conducting and evaluating GHD is a consideration of challenges and barriers concerning national action.
Pearlman et al. [21]	–	2016	Science & Diplomacy	Global	GHD, Cancer, Multi-stakeholder collaborations, NCDs	case study	Document review and evidence-based survey	overcome many barriers with Effective partnership and careful coordination
Wipfli & Samet [41]	–	2012	Tobacco Control	Global	GHD, FCTC, International Negotiations	Commentary and perspectives	Extending the lessons learned from tobacco control to NCD control	The collective response to NCDs should centralize on generating multisectoral evidence about the transnational factors influencing the rise in NCDs and their risk factors.
Juma et al. [42]	Kenya, South Africa, Cameroon, Nigeria, and Malawi	2018	BMC Public Health	National/ Regional	Multi-sectoral Action	multiple case study	Document reviews and key informant interviews, McQueen’s framework for intersectoral governance	The need for mechanisms including approaches to capacity building and resource production to be able to take multi-sectoral measures in policy development, implementation and monitoring of NCD results.
Dain [43]	–	2018	International Journal of Health Policy and Management	Global	Civil Society Networks, Coalition Building, Advocacy, Governance, Human Sustainable Development	Commentary and perspectives	Shiffman’s ‘Four Challenges that Global Health Networks Face’	NCDA’s* advocacy has contributed to the call for global political commitment.
Russell et al. [44]	–	2018	Global Public Health	Global	GHD, E-cigarettes, FCTC, International Law	Original study	Ethnography method	One of the important tasks of the FCA is to prepare policy brief on key issues.
Frech [45]	Latin America	2018	Journal of Global Oncology	Regional/ Global	Cancer Research and Control, Partnerships and Diplomacy	Commentary and perspectives	Qualitative	The need for high-level cooperation (the President’s commitment), the sharing of information to support the efficient use of limited resources, the prevention of repeated attempts, and the synergy of current investments in specific countries are essential.
Beaglehole [46]	–	2011	Lancet	Global	UN* High-Level Meeting on NCDs, Global Crisis, Need for Global Response, International Cooperation, Monitoring and Accountability	Commentary and perspectives	Report- providing evidence for the realities of the NCD situation, summaries key messages for heads of state and governments.	Long-term success requires inspiring and committed national and international leadership, improving primary health care, effective use of existing resources, new financing methods.
Nishtar et al. [47]	–	2018	Lancet	Global	WHO Independent High-level Commission on NCDs, National Response to NCDs, International Cooperation	Commentary and perspectives	This report represented rich and diverse views and perspectives.	Ensureing implementation through legislation, regulation and standards or investment. Health in all policies, approaches of the whole government, the whole society and intersectoral approaches must be taken in the field of NCDs actions. Need technical support, training, practical research and capacity building initiatives.
Samuels & Hospedales [48]	CARICOM	2011	West Indian Medical Journal	Regional/ Global	Heads of government, UN High-Level Meeting on NCDs	Commentary and perspectives	Report	commit to strengthening systems and incrising resources, endorse and implement the commitments made and identify and support leadership for sustained action and accountability for these initiatives.
Hatefi et al. [49]	–	2018	Bulletin of the World Health Organization	Global	Global Susceptibility to NCDs, Accountability	Commentary and perspectives	Perspectives on rational response to global health risks	The main response to NCDs must happen downstream at the country level.
Wickramasinghe et al. [50]	Lebanon, Morocco, Sudan, and Yemen	2018	Global Health Action	National/ Regional	Multisectoral Action, National-level stakeholders	original study	Structured interviews with key stakeholders	Achievement to national multisectoral action plans development through collaboration and good technical support.
Mendis [51]	–	2010	British Medical Bulletin	Global	Policies to Support Regulatory, Legislative, Intersectoral Action	Commentary and perspectives	Qualitative review	Needed to develop innovative approaches for revenue generation for prevention and control of NCDs. Adapted agenda concering the context of contries.
Maher & Sridhar [52]	–	2012	Journal of Global Health	Global	Global Fight Against NCDs, global health policy communities, political leaders	discipline configurative case study	Qualitative -Shiffman’s 2009 political priority framework	Engaging the diverse actors for the global proliferation of NCDs.
Gneiting & Schmitz [53]	–	2016	Health Policy and Planning	Global	Advocacy, Political Factors, Network Formation and Evolution in International Health Governance	Original study	In-depth qualitative analysis, in-depth examination of social and political processes with a paired comparison	global health networks (individuals to a global coalition of membership) are engaging in advocacy on a given health problem.
Magnusson [54]	–	2007	Globalization and Health	Global	Global Health Governance	Commentary and perspectives	Report	Needed to broader framework of reference for lifestyle-related NCDs
Battams & Townsend [55]	–	2018	Critical Public Health	Global/ nation	trade policy, policy coherence, social determinants of health, advocacy	original study	Interviews with key actors working across trade and health sectors	Support for advocacy coalitions operating basedn upon trade and geopolitical interests. Lobbying trade policy actors proactively and benefit from linking with global advocacy networks as a way to counter the power and resources of industries with NCD risk areas.
Kirton et al. [56]	CARICOM	2018	Pan American Journal of Public Health	Regional/global	Port of Spain Summit Declaration, global and regional action	Original study	Using data from published literature, primary documents, and semistructured interviews (a method developed by the University of Toronto’s Global Governance Program)	requirement to embed NCDs in a whole-of-global-governance approach, monitor implementation annually, develop transregional partnerships, engage civil society and support regular regional and global summits
Greaves et al. [57]	CARICOM	2018	Pan American Journal of Public Health	Regional	Port of Spain Summit Declaration, Health communication	Commentary and perspectives	Report and review	the NCDs advocate should be considered as knowledge broker performing tasks related to effective knowledge transfer, networking and capacity building
Chattu et al. [58]	CARICOM	2019	Health Promotion Perspectives	Global	Port of Spain Summit Declaration, outcome of GHD	review	systematic review	Impact of the NCDs regional summit declaration on global attention to the Prevention and Control of NCDs.
McBride et al. [59]	–	2019	BMC Public Health	Global	GHD, Soft power, Global health agenda-setting, SDGs*, BRICS, G7, G20	Original study	Content analysis to review the health ministerial communiqués issued by the political clubs after the SDGs were adopted at the UN General Assembly of September 2015	The global health leadership of the BRICS, G7 and G20 represents an exercise of soft power and GHD on NCDs and their risk factors.
Collins et al. [60]	–	2019	The bmj	Global	development cooperation, global action,	Commentary and perspectives	Report and review	Development South-South and triangular cooperation beyond North-South development assistance.

^*^ noncommunicable diseases (NCDs), global health diplomacy (GHD), United Nations (UN), World Health Organization (WHO), non-governmental organizations (NGOs), The Caribbean Community (CARICOM), Framework Convention on Tobacco Control (FCTC), noncommunicable diseases alliance (NCDA), Sustainable development goals (SDGs)

In Table 2, five themes are identified using the Blouin and Dubé’s (2010) analytical framework for examining negotiations: (1) the specific problem requiring global collective action, (2) key actors, (3) their interests in the problem, (4) potential negotiation process, and (5) potential scenarios for collective action. Besides, 46 sub-themes related to GHD on NCDs are identified (Table 2).

**Table 2:** Analytical framework for examining negotiations

Themes	Subthemes
(1) The specific problem requiring global collective action	- Globalization of marketing and advertising strategies - Sanctions, including financial, travel and trade restrictions - Multinational companies undermining the regulatory authority of national governments through public relations and lobbying strategies, and the need to develop coordinated strategies and diplomatic initiatives to address the multinational nature of the problem - GHD on obesity and tobacco control requires stronger engagement with developing countries - The need for collective action against NCDs given the current migration and travel patterns - Smuggling - Trade liberalization, including a reduction in trade barriers and prices to increase competition in the international market - New global challenges such as climate change
(2) Key actors	- United Nations and associated agencies and groups, including the WHO and the Independent High-level Commission on NCDs, and groups and important political clubs such as G7, G8/ BRICS - the Food and Agricultural Organisation (FAO) - the World Bank - the World Trade Organisation (WTO) - the Codex Alimentarius Commission - Heads of government - National governments and ministries (e.g. health, foreign policy, education, finance, labor, etc.) - Regional coalitions of countries - International Diabetes Federation - World Heart Federation - Asia-Pacific Economic Cooperation (APEC) - Health advocacy groups, NGOs, and humanitarian organizations - International professional associations - Commercial actors (e.g. pharmaceutical companies, a variety of businesses involved in the production, processing, distribution, marketing, and sales of food and beverage) - Civil society organizations and coalitions (e.g. Nutrition Foundation, National Center for Chronic Disease Prevention, National Cancer Institute) - Healthcare regulatory agencies - Parliamentary Committee for Prevention and Control of NCDs - National Inter-ministerial Tobacco Control Committee - NCD Alliance, Framework Convention on Tobacco Control, and Union for International Cancer Control - Research centers and the academia - The medical community - The private sector - Patients and their families - International donors
(3) Actors’ interests in the problem	- UN and WHO: technical support, non-binding recommendations and policy advice, international legal obligations and laws, advocacy - Groups and political clubs such as G7, G8/ BRICS: Supporting reforms, develop framwork for regulation, support growth and development across globe, Strengthen international cooperation - Governmental actors: governments represent power in diplomacy, while NGOs represent ideas and knowledge - NGOs: monitoring, lobbying, brokering knowledge, offering technical expertise, and fostering inclusion; NGOs act as a catalyst to the process of developing policies and drive it by including stakeholders, offering technical expertise, and providing financial assistance; multisectoral partnerships aimed at capacity-building and strengthening health systems - Industrial actors: reduced market share and profits due to measures to limit tobacco use - Unions: advocacy of NCD Alliance to trigger a global policy response, the role of Framework Convention Alliance (FCA) in agenda-setting for tobacco control - Policy champions: strong supportive role
(4) Potential negotiation process	- Scientific and technical capacity building - Preparation for negotiations, mobilization of civil society organizations, dialogue with industry, consultation with experts, and sharing of information among national health agencies - Entering the negotiating forum - Monitoring, lobbying, brokering knowledge, offering technical expertise, and fostering inclusion
(5) Potential scenarios for collective action	- Drawing attention to issues that are not yet on the GHD agenda or are largely overlooked - Political leadership, strong mobilization, and advocacy from well-organized groups globally are crucial in triggering and sustaining a global policy response such as an international treaty - A monitoring role that involves publicizing the diplomatic process

Given the importance of mapping actors, their interests, and structural characteristics related to them, interactions in the international arena on NCDs are divided into three categories using the GHD Pyramid: (1) core diplomacy, (2) multi-stakeholder diplomacy, and (3) informal diplomacy. Actors, tools, contents, and contexts were extracted at each of these three levels (Table 3).

**Table 3:** Prevention and control of NCDs at different levels of the GHD Pyramid

	Actor	Tool (Process)	Content	Context
Core Diplomacy	- UN - WHO - the Food and Agricultural Organisation (FAO) - the World Bank - the World Trade Organisation (WTO) - the Codex Alimentarius Commission - Independent High-level Commission on NCDs - Heads of government - International professional associations - International Diabetes Federation - World Heart Federation - Asia-Pacific Economic Cooperation (APEC) - The Caribbean Community (CARICOM)	- Policy brief - Technical support - Exchange of experiences at the international level - Knowledge generation International legal obligations and laws - non-binding recommendations and policy advice - advocacy	- UN High-Level Meeting on Prevention and Control of NCDs - WHO Global NCD Action Plan - WHO Global Monitoring Framework for NCDs - Recognition of NCDs as a major challenge to sustainable development - Alliances, treaties, and other agreements - FCTC - Regional Action Plan for Prevention and Control of NCDs - Port of Spain Summit Declaration “Uniting to Stop the Epidemic of Chronic NCDs” - WHO Global Strategy on Diet, Physical Activity and Health - Global Code of Practice on the Marketing of Unhealthy Food and Beverages to Children - Resolutions WHA63.14: Marketing of Foods and Non-alcoholic Beverages to Children - MPOWER Package - Best Buys - 2017 WHO Cancer Resolution - WHO Mental Health Action Plan - Montevideo Roadmap 2018–2030 on NCDs as a Sustainable Development Priority - The Paris Declaration on Aid Effectiveness	- Need for network formation at regional and international levels - Lack of resources at national and international levels - Effect of the political will of politicians - The instrumental use of GHD to achieve other foreign policy and diplomatic goals - Differences in wealth, incidence and prevalence of NCDs, and method of disease control
Multi-stakeholder diplomacy	- Ministry of Health - Ministry of Industry - Ministry of Agriculture - Ministry of Education - Ministry of Urban Development - Ministry of Sports - Environmental organizations - Media - The academia - Commercial actors - Civil society organizations and coalition - Healthcare regulatory agencies - Nutrition Foundation - National Center for Chronic Disease Prevention, National Cancer Institute - Parliamentary Committee for Prevention and Control of NCDs - National Inter-ministerial Tobacco Control Committee	- Advocacy - Scientific research - Health in All Policies - Clear guidelines to strengthen coordination mechanisms - Whole-of-government approach - Publication of a newsletter, shaming, and symbolism - Media advocacy to influence policy positions of countries during negotiations - Holding scientific conferences	- National Document on Prevention and Control of NCDs - Agreements between ministries - National Plan for Tobacco Control - Development plans	- Advocacy varies across different governments and ministries (different cultures) - Programs developed to change systems and cultures (e.g. lifestyle changes) require sustainable medical and political leadership - A single plan is not responsive for all countries and different contexts - Significant difference between low-income countries and the global context - Political obstacles, including sanctions - Geographical location, communication, and health infrastructure - Language barriers and the need to translate tools to native languages - Health-related socioeconomic factors are not limited to the public health sector alone and exist in other sectors of the health system, highlighting the need for multi-sectoral action - Poverty and social factors affecting health - Implementing changes for cancer control that are tailored to a specific context, society, and culture - NCDs threaten economic and human development
Informal Diplomacy	- NCD Alliance - Research centers and academic associations - The medical community - The private sector - Patients and their families - International donors - NGOs - Health advocacy groups, NGOs, and humanitarian organizations - National Cancer Institute	- Media - Campaigns against alcohol and tobacco use - National Campaign for Tobacco Control and Smoking Cessation - Specific activities such as games for the elderly - World No Tobacco Day	- NCD Advocacy Document	- Understanding the historical context of negotiations - Any country is several markets rather than one, with a wide variety of marketing types

The key actors are identified at different levels of diplomacy: Core diplomacy: UN and associated agencies and groups, including the WHO and the Independent High-level Commission on NCDs, heads of government, international professional associations, International Diabetes Federation, Union for International Cancer Control, and World Heart Federation; Multi-stakeholder diplomacy: Asia-Pacific Economic Cooperation (APEC), national governments and ministries (e.g. health, foreign policy, education, finance, labor, etc.), commercial actors (e.g. pharmaceutical companies, a variety of businesses involved in the production, processing, distribution, marketing, and sales of food and beverages), civil society organizations and coalitions (e.g. Nutrition Foundation, National Center for Chronic Disease Prevention, National Cancer Institute), healthcare regulatory agencies, Parliamentary Committee for Prevention and Control of NCDs, and National Inter-ministerial Tobacco Control Committee; Informal diplomacy: NCD Alliance (NCDA), Framework Convention on Tobacco Control (FCTC), and Union for International Cancer Control, research centers, the academia, the medical community, the private sector, patients and their families, international donors, and health advocacy groups, NGOs, and humanitarian organizations.

Critical information are discussed in the content of some declaration and agreement for raising global challenges to GHD and negotiation (Table 3). For example The Paris Declaration on Aid Effectiveness has a leading role in raising awareness of climate change on the international stage and advocating for strong climate action for new global challenges such as rising food and climate change and Port of Spain Summit Declaration helped to rise NCDs agenda in global setting.

## Discussion

The purpose of this Systematic review was to draw lessons for strengthenning linkages with a wide range of actors and stakeholders from the GHD literature regarding the prevention and control of NCDs. GHD is focused on international negotiation that includes a wide range of processes, from finalizing agreements between multilateral or bilateral aid donors and recipient countries to the processes of making binding and non-binding international agreements in health or related to health. Between 2007 and 2019, 30 articles have been published on GHD for NCDs.

Five themes, i.e. (1) the specific problem requiring global collective action, (2) key actors, (3) their interests in the problem, (4) potential negotiation process, and (5) potential scenarios for collective action and 41 sub-themes on GHD in prevention and control of NCDs were identified. Moreover, given the importance of collaboration on NCDs in the international arena, actors were categorized into three groups based on the GHD Pyramid: (1) core diplomacy, (2) multi-stakeholder diplomacy, and (3) informal diplomacy.

Seven specific problems that require global collective action were identified: the globalization of marketing and advertising strategies, sanctions, multinational companies undermining the regulatory authority of national governments, the need for stronger engagement with developing countries for GHD on obesity and tobacco control, current migration and travel patterns, trafficking, and trade liberalization. International commitments have changed the dynamics of countries’ domestic policies. For example, multinational tobacco companies undermine the regulatory authority of national governments through public relations and lobbying strategies [61]. Due to the asymmetry of resources between large global tobacco firms and the governments of small countries, this problem is specifically acute in developing countries [36]. The adoption and implementation of tobacco control measures have strengthened the position of public health advocates against pressure from multinational tobacco companies [36].

One of the lessons learned from tobacco control is the need to focus on the upstream factors in the NCD pandemics such as multinational companies and globalized advertising and promotion of unhealthy products. Tobacco control activity at the global level is affected by the actions of the tobacco industry, increasing globalization of aggressive marketing, and changing social norms regarding smoking. As a result, it is difficult for any country to control tobacco use within its borders and a collective response is necessary. Policies focused on increasing price, decreasing access, restricting advertising and improving labeling could be applied to products that promote NCDs, especially alcohol and processed foods that are high in sugar and fat [41].

The complexity and costs associated with effective management of NCDs require the involvement of a diverse group of actors. Given the increased pressure faced by developing countries with unprepared systems and economies, diverse partnerships may be a more important component in effective prevention and management of NCDs [30]. GHD researchers have studied international negotiations as a “two-level game” at national and international levels. At the international level, governments seek to maximize their ability to meet domestic pressures, while minimizing the negative consequences of foreign development. At the national level, domestic groups pressure the government to adopt favorable policies for their interests, and politicians seek power by building coalitions among those groups [62]. There is power asymmetry in international negotiations. This is exacerbated in the context of NCDs and GHD since health ministries and agencies are often less powerful within their governments. Two strategies to overcome this challenge are building coalitions and preparing for negotiation [30].

Governments must be the key stakeholders in policy development and provide leadership for implementation, monitoring, and evaluation through a multi-stakeholder platform. The government may decide to cooperate with other sectors and stakeholders, but it is in the best position to set the direction and overall strategy for achieving public health goals [63]. There are differing views within the government about the specific positions and strategies the country should take during negotiations. Creating an inter-ministerial committee is a useful way of using these different perspectives in international negotiations. The history of health-related trade negotiations indicates the importance of these institutional mechanisms for intersectoral collaboration [64]. After setting a clear negotiating agenda at the national level, the most conventional form of GHD takes place, i.e. formal negotiations between parties. In international negotiations, parties with different preferences need to exchange concessions. One party concedes to the preferences of the other party and, in exchange, ensures that its priority is reflected in the final text. In these negotiations, building coalitions with like-minded parties is a useful strategy for smaller countries that seek to achieve their objectives [30].

In the context of NCDs, a key aspect of GHD is the variety of nonstate actors that must be engaged with at different parts of the process, and it is crucial to understand the incentives of these actors pertaining to the costs and benefits of participation [34, 65, 66]. Civil society organizations must be involved in international negotiations on public health issues and must focus on providing and sharing relevant scientific information with national delegates. All the delegates and FCA participants in the FCTC argued that, at the beginning of the Intergovernmental Negotiating Body (INB) sessions, there were significant differences in the knowledge of national delegates of tobacco use and tobacco control. The FCA has helped close this gap by providing and sharing information with the delegates [37]. It is the actors involved in a negotiation process that determine whether to place an issue on the national and international agenda and whether to create alternatives for its effectiveness. It is thus necessary for NCD stakeholders to come together within and among countries to advocate for their needs and priorities [21].

Six key sub-themes were identified for the theme “actors’ interests in the problem”. WHO recommendations are non-binding, but represent the organization’s official policy and reflect its norms and standards. Moreover, these recommendations indicate successful GHD. However, successful international agreements are not always implemented at a national level, and thus, they may not bring about the desired health benefits [40]. A weakness of the UN Declaration on the High-Level Meeting on NCDs in September 16, 2011, is that it was lacking in targets, funds, and activities. An important determinant in the relatively weak outcome of this exercise in GHD is the weak presence of advocacy groups and activists regarding NCDs. An important lesson from successful GHD in the past is the key role of civil society organizations in ensuring a strong collective response. This factor was critical to the success of FCTC negotiations [46].

It is necessary to strengthen governance and coordination structures across different sectors and levels to ensure that all relevant sectors are involved in NCD prevention measures. Countries must use a strong advocacy and communications strategy on multi-sectoral action for NCD prevention in order to increase NCD awareness among various sectors and resolve conflicts. Sustainable joint financing mechanisms are needed for the effective implementation of these actions. In addition, countermeasures must be employed to prevent industries from obstructing the implementation of NCD prevention measures [42]. Conflict of interest can hinder or halt policy development or participation of different sectors. One of the negative effects of actors’ interests is industry interference with the process of policy development and implementation, especially the tobacco industry, which has interfered with the policy process in almost every country in LMIC. Market forces contribute to an increase in NCDs. For example, pandemics related to obesity and tobacco and alcohol use are mainly due to the successful marketing of unhealthy products. This outcome is a serious justification for government intervention through regulatory and legal responses.

NCD control requires political champions for advocacy. The most important outcome of the new diplomatic attention to NCDs is the platform provided for greater coordination on NCDs among the UN, national governments, and civil society. One of the key challenges in NCD prevention and control is the complexity of these diseases, as they are caused by a variety of risk factors and are associated with various agents, from the international level (e.g. multinational fast food companies) to the local level (e.g. unwalkable streets). In addition, not all NCDs are preventable. Due to these complexities, it is difficult to specify their targets and funds. The global response to NCDs must focus on generating multisectoral evidence about the transnational factors that contribute to the rise in NCDs and the potential impact of policies proposed to control them [41].

As for the theme “the potential negotiation process”, four sub-themes were identified, including scientific and technical capacity building, preparation for negotiation, entering the negotiating forum, and monitoring, lobbying, brokering knowledge, offering technical expertise, and fostering inclusion. GHD is a political process based on the intersection of health, foreign policy, and trade that can take place at bilateral and multilateral levels through political negotiations [66, 67].

Therefore, it is first necessary to strengthen the scientific and technical capacities of health attachés and develop diplomats for the GHD platform. Preparation for negotiations by mobilization of civil society organizations, dialogue with industry, consultation with experts, and sharing of information among national health agencies are crucial steps before entering negotiations [36]. Moreover, to address many global public health issues in which some countries put political expediencies and their interests above collective global interests, one strategy is to hold countries accountable by publicizing their positions and reward positions that support a strong treaty [37]. GHD always takes place through national politics. Therefore, the successful conclusion of international negotiations is not the end, but part of a process and GHD must be conducted and evaluated by considering the barriers and issues concerning national action [40].

Three sub-themes were identified for the theme “potential scenarios for collective action”: First scenario: Drawing attention to issues that are not yet on the GHD agenda or are largely overlooked. GHD is the process of negotiated collective action for global health that can eventually lead to new forms of global health policy and governance for tackling global health challenges. It is clear that the development of national policies and FCTC negotiations have clearly interacted. Once Canada adopted large graphic health warnings, Thailand, Brazil, and the European Union followed suit [68]. Until recently, the field of GHD on NCDs was primarily WHO-centric. A key challenge was to bring NCDs beyond the traditional health forum and that is why the UN High-Level Meeting is regarded as a great opportunity. When deciding to engage in GHD for a problem identified during the agenda-setting phase, an important consideration for policymakers is the impact of the media on the political agenda. The media is deemed to play a greater role in putting issues on the foreign policy agenda than the domestic policy agenda [69].

Second scenario: Political leadership, strong mobilization, and advocacy from well-organized groups globally are crucial in triggering and sustaining a global policy response such as an international treaty. Success in tackling the NCD crisis depends on strong and effective leadership. A strong leader can act as a catalyst and bring about change [70]. Moreover, NGOs and civil society have provided significant support for policy development and the interaction of various actors. NGOs can act as a catalyst to trigger the policy process and drive this process by coordinating the entry of stakeholders, offering technical support, and providing financial assistance. In addition, coalitions and networks may be formed ton ensure that implementation of certain policies will continue. These networks tend to engage various sectors in this process. Professional associations can also become part of advocacy and coalition networks [41].

Third scenario: A monitoring role that involves publicizing the diplomatic process, such as exposing the position of countries in documents distributed to diplomats and on the Internet for the general public. There is an established Monitoring and Evaluation for Port of Spain declaration which is robust and no other NCD policy has such a mechanism on a periodic basis. Monitoring of the Declaration was cunducted by CARICOM and the Pan American Health Organization (PAHO) and evaluating was cunducted by the University of the West Indies [67, 71].There is an urgent need for advocacy to raise NCD awareness at the national and international levels as a development problem, not just a health problem. In addition, increasing the capacity for policy research and implementation is needed in all countries [46].

## Conclusion

This paper has highlighted the role of global health diplomacy in all three levels for preventing and controlling of NCDs. There is an urgent need for advocacy to raise NCD awareness at the national and international levels as a development problem, not just a health problem. In addition, increasing the capacity for policy research and implementation is needed in all countries. The development and adoption of a global policy to tackle the rise in chronic diseases in developing and developed countries requires policymakers that engage in GHD. Successful developments in global health policy depend on the performance and respectful relationships of stakeholders, and global health diplomats need to have an understanding of the complexities of institutional structures and the functional relationships among international institutions involved in health. A successful and sustainable plan for tacking NCDs entails partnerships among national governments, the private sector, and civil society at international, national, and local levels.

## Data Availability

Not applicable.

## Abbreviations

NCDs: Noncommunicable diseases
GHD: Global health diplomacy
UN: United Nations
WHO: World Health Organization
LMIC: Low and middle-income countries
NGOs: Non-governmental organizations
APEC: Asia-Pacific Economic Cooperation
PAHO: Pan American Health Organization
CARICOM: The Caribbean Community
FCTC: Framework Convention on Tobacco Control
NCDA: Noncommunicable diseases alliance
SDGs: Sustainable development goals
